# Flux of granular particles through a shaken sieve plate

**DOI:** 10.1038/srep09880

**Published:** 2015-06-09

**Authors:** Pingping Wen, Ning Zheng, Junwei Nian, Liangsheng Li, Qingfan Shi

**Affiliations:** 1 School of Physics, Beijing Institute of Technology, Beijing 100081, China; 2 Science and Technology on Electromagnetic Scattering Laboratory, Beijing, 100854, China

## Abstract

We experimentally investigate a discharging flux of granular particles through a sieve plate subject to vertical vibrations. The mean mass flux shows a non-monotonic relation with the vibration strength. High-speed photography reveals that two stages, the free flight of the particles’ bulk over the plate and the adhesion of the particles’ bulk with the plate, alternately appear, where only the adhesion stage contributes to the flow. With two independent methods, we then measure the adhesion time under different vibration conditions, and define an adhesion flux. The adhesion flux monotonically increases with increasing vibration strength. By rescaling the adhesion flux, we find that the adhesion flux is approximately determined by the peak vibration velocity of the shaker. The conclusion is examined with other sieve geometries.

Investigations on the flow properties of bulk solids, such as ores, grains and pharmaceuticals, have a wide application in conveyance, processing and storage of these materials^1^,^2^,^3^. In nature, many important phenomena like avalanches, sandstorms, desert migration, and even traffic flow can be related to the bulk solids flow, or granular flow^4^,^5^,^6^. Among these studies, granular flow from a hopper is one of the most prolific topics due to its importance in industrial silos and hoppers, as well as the relevance in the significant statistical mechanics problem of a fluid flow through a hole^7^,^8^,^9^.

In contrast to the flow of a viscous fluid, granular flow exhibits many exotic behaviors. For example, the discharge rate remains constant with decreasing depth of granular particles above the outlet. The most acceptable explanation is that the side walls of a hopper have burdened the weight of the materials by means of the friction force, which results in a pressure saturation at the bottom^10^,^11^,^12^. Aguirre el at even suggest that the flux of particles from an orifice is independent on the bottom pressure and the velocity of the particles near the orifice is the crucial parameter to determine the discharge rate^13^,^14^. When the orifice is smaller than some critical size, granular flow always halts due to the spontaneous clogging near the orifice^15^,^16^,^17^. To reduce the clogging probability, several approaches have been proposed. Inserting an obstacle above the outlet can effectively reduce the pressure above the outlet, and thus prevent the generation of blocking arches^18^,^19^. Another method more frequently used is shaking, or vibration. By introducing an external excitation, the blocking arches become unstable and are readily broken, and then the flow resumes^20^,^21^,^22^.

Although the clogging often needs to be unfrozen in practice by vibrations, to our knowledge, only a few researches concentrated on the discharge rate or flux from vibrating hoppers, or silos^20^,^23^,^24^,^25^. Furthermore, the experimental system in previous studies only possesses one hole as the discharging outlet, and the diameter of the hole is usually larger than the critical diameter, in which the granular flow persists even without the external vibration. If the particles are spherical, the critical diameter of the outlet has to be about 4–5 times of the diameter of a single particle. Therefore it is necessary to consider a limiting case where the size of the outlet is much smaller than the critical diameter to explore some underlying physics. Compared with the previous experimental systems, our experimental setup is considerably different. There are hundreds of identical holes instead of a single hole, and these are placed on the flat bottom of a container for the granular discharge. Moreover, the diameter of each hole is much smaller than the critical diameter, only allowing one single particle to pass through each time. Without external vibrations, flow clogging always occurs. However, within the range of the vibration conditions, clogging never appears in our vibrating container with a porous, flat bottom which can be seen as a sieve plate. Actually, another reason for choosing such sieves is that shaken sieves are more routinely used to separate bulk materials in industrial and agricultural productions.

In this paper we study the mean mass flux of granular particles through a sieve plate in a vertically vibrated container. To explain the complex non-monotonic dependence of the flux on the vibration strength, we apply a high-speed camcorder to record the motion of the particles near the sieve plate. By analyzing the images, it is found that two stages, the free flight of the particles’ bulk over the sieve plate and the adhesion of the bulk on the sieve plate, alternate. The flow only occurs during the adhesion stage. We then measure the adhesion time by two independent methods, and calculate another flux, i.e. adhesion flux, by just taking the adhesion time into account. Thus we find a monotonic relation between the adhesion flux and the vibration strength. On the basis of these experimental results, we analyze the dual effects of the vibration strength on the flux. Finally, we rescale the adhesion flux to find the adhesion flux is nearly determined by the peak velocity of the shaker.

A sketch of the experimental setup is demonstrated in Fig. 1. A shaker supplies the vertical vibration to fluidize the particles in order to continue the flow, and an electronic scale weighs the outflowing particles from the sieve plate. The experimental details are described in the methods section.

## Results and Discussion

### Mean mass flux

In our experiment, the alumina particles are poured into the shaken container at one go. Fig. 2(a), as an example, shows the mass of the outflow as a function of time for different accelerations Γ with a given frequency. A nearly linear relation between the mass and time is shown. To determine the mean mass flux, the transient parts at the start and end of *m*(*t*) curve are first removed. The flux 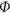 can be obtained from the slope of linear fits to the remaining part of *m*(*t*) curve, usually within the range of 70–430 g. We also compare the flux which is extracted from the linear fits to the whole *m*(*t*) curve, namely, 0–500 g. The two fluxes differ slightly, but the result in Fig. 2(b) remains unchanged qualitatively in either flux. Since the vibration parameters *f* and 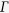 can change the flux, correspondingly the curves of the flux 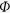 as a function of the vibration strength 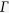 at different frequencies are plotted, as shown in Fig. 2(b). At high frequency, such as *f* = 150 Hz, the flux shows a monotonic dependence on the vibration strength. The flux at the high frequency steadily increases with increasing 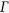 (i.e., the harder we shake a sieve, the more the particles flow out per unit time). However, the measurement at the high frequency differs from previous results which showed a measured flux as a decreasing function of the vibration strength^24^,^25^. This contradiction can probably be traced to the differences in these experimental systems. Obviously, without the vibrations, the flux of particles does not exist in our system.

However, at low frequencies the flux decreases with some stronger vibration. The flux curve exhibits complicated non-monotonic dependences on the vibration strength. The curve at *f* = 30 Hz, for instance, first increases at weak vibration strength and reaches a peak at 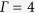, and then drops when 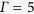, and eventually rises again after 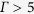. The dissimilar responses of the flux to the vibration strength at different frequencies imply that a competitive mechanism could exist, which affects the flux in a shaken granular system. Thus, it is necessary to observe the flow process in detail.

### Flight and adhesion stage

When inputting external energy into a static granular pile, the pile becomes fluidized and exhibits more features resembling a fluid. Generally, a fluid flow is treated as a continuous process, which is also applicable in the issue of the granular flow through an immobile hopper without clogging. However, the granular pile in our experiment periodically separates from the sieve plate and experiences a period of free flight when the vibration acceleration is larger than the acceleration due to gravity *g*. To reveal the details, a piezoelectric pressure transducer (PCB 208C01) firmly embedded to the center of the sieve plate is used to measure the normal force exerted by the granular pile. The area ratio of the transducer to the whole plate is less than 5%, which ensures that the influence of the transducer on the flow is insignificant. Fig. 3(a) as a typical example shows the measured signal by the transducer as a function of time. During the free flight, the pile loses contact with the sieve plate, and thus the pressure on the transducer vanishes. When the pile collides with the sieve plate, the pressure dramatically increases, see the sharp pulses in Fig. 3(a). The width of each peak corresponds to the adhesion time 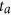, and the time interval between the two peaks is the free flight time 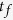.

To examine the validity of the free flight and adhesion time extracted from the pressure signal, we make use of another method to independently measure the two times. A high-speed digital camcorder is applied to record the motions of the particles around the sieve plate. Fig. 3(b) uses a series of successive snapshots in one vibration cycle of the shaker to illustrate the flow process. The free flight period of the granular pile can be clearly manifested, as shown from first photograph *t* = 105 *ms* to fourth photograph *t* = 152 *ms*. During the period, the whole pile thrown in the air completely loses contact with the sieve plate and only a few particles stay on the plate. Consequently, almost no particles can pass through the upper surface of the plate (indicated by a red line in the second photograph) to form the granular flow as the granular pile is flying, which contributes nothing to the flow (the flow shown in the photograph actually is the continuation from the last adhesion stage). Subsequently, the granular pile falls and just collides with the sieve plate (see the photograph *t* = 152 *ms*), and then the particles above and near the plate pass through the holes (see the photograph *t* = 167 *ms*). The flow thus resumes until the next free flight of the granular pile (see the last photograph). It is noteworthy that both times measured by the two methods are nearly the same. According to the experimental observation, the flow process can be divided into two stages: the free flight stage where the granular pile detaches from the sieve plate and no flow through the upper surface of the plate can occur; the adhesion stage where the pile moves together with the plate, and the bottom particles of the pile pass through the sieve plate, forming a mass flow. The repeated pausing and resuming of the flow constitute an intermittent flow when subjected to a vertical vibration. The discontinuity of the flow presumably contributes to the intricacies of the relationship between the measured mass flux and the vibration strength. It should be noted that Wassgren *et al.* in their simulations also found the discharge flux as a function of phase angle during the vibrating cycle at different vibrating frequencies^24^.

### Adhesion time fraction χ

As the free flight stage periodically alternates with the adhesion stage, the whole flow process can be conveniently divided into two processes; namely the free flight and adhesion process. Correspondingly, the total mass of the whole outflow 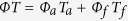 consists of two parts, the outflowing mass, 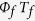, from the free flight; and the outflowing mass, 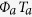, from the adhesion stage, where 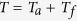, 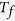 and 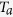 are the sum of the free flight and adhesion time in each vibration cycle, respectively. As analyzed before, only the adhesion process contributes to the flux. It makes sense that the flux 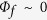 in the free flight stage. According to the equation 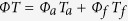, we naturally derive an effective flux, namely adhesion flux 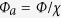 with the adhesion time fraction 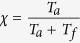.

In the experiment, two independent methods are used to measure the adhesion time fraction 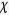. Here the adhesion time and free flight time are mainly extracted from the videos recorded by the high-speed camcorder. We have observed an excellent periodicity in the free flight and adhesion stage, that is, approximately both the durations are constants for given vibration conditions. Thus, twenty cycles of the free flight and adhesion stage are arbitrarily adopted to calculate the adhesion time fraction 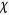. To justify the generality in the selection of these cycles, the adhesion time fraction 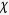 is examined when the discharging mass is at 100 *g*, 250 *g* and 400 *g*, respectively, as shown in the inset of Fig. 4(a). The overlapping curves indicate that the adhesion time fractions measured at different masses are almost the same when the vibration condition is given, which further verifies that the adhesion time fraction from 20 cycles to represent the adhesion time fraction in the whole process is sufficient.

Fig. 4(a) shows the adhesion time fraction 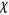 as a function of the vibration strength 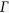 at various frequencies. The adhesion time fraction monotonically reduces with increasing vibration strength. It is because for a larger vibration strength, the takeoff velocity of the granular pile is larger as well, which means it takes more time for the pile to fall back to impact on the sieve plate. Compared with high frequencies, the fall tendency of these curves in Fig. 4(a) becomes more abrupt for low frequencies. At low frequencies, it has been observed that the granular pile contacts with the plate in every cycle of the shaker for small 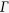, but the pile contacts with the plate in every two cycles of the shaker as 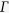 increases. We have even observed that in three shaker cycles only one contact happens at *f* = 15 Hz, 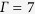. At high frequencies, the granular pile can always touch the sieve plate in each cycle over the given range of the vibration strength. Thus the adhesion time fraction falls more rapidly for lower frequencies. For the frequencies below 40 Hz, the adhesion time fraction reduces quickly to 0.3 or even smaller at 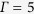. In contrast, the adhesion time fraction just decreases to 0.7 for the frequencies above 70 Hz with the same vibration strength.

### Effective flux: time-averaged adhesion flux

With the adhesion time fraction 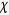, an effective flux is introduced to account for the complex behavior in Fig. 2(b). The effective flux also called adhesion flux, 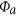, is defined as 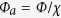, where 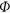 is the mean mass flux mentioned before. The adhesion flux 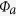 is plotted as a function of the vibration strength 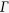, as shown in Fig. 4(b). These curves show that the adhesion fluxes monotonically increase with increasing vibration strength over the range of the vibration frequency. Furthermore, the fluxes show a nearly linear dependence on the vibration strength except for the situation in which the vibration is very weak.

It has been known that only the adhesion stage can contribute to the flow, and the free flight of the granular pile where the flux 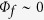 leads to the intricacy in the relation of the mass flux 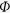 and the vibration strength 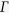. When the free flight time is removed from the total time, the complex relation disappears. We consider the vibration strength presumably induces two opposite effects, namely the fluidization extent and the free flight of the granular pile, which influence the mass flux 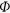 simultaneously. As the vibration strength rises, the granular pile can acquire more energy from the shaker and create stronger fluidization, which favors the flux. On the other hand, with the increase of the vibration strength, the duration of the free flight can last longer, which weakens the flux. The complex relationship between mass flux and vibration strength is the result of the competition of these two opposite effects.

To explore more underlying physics in the adhesion flux, the experimental data in Fig. 4(b) have been rescaled to show how the vibration frequency *f* depends on the rescaled flux 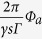 in Fig. 4(c). Here, *s* is the area of the sieve plate, 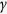 is the porosity of the plate, and 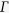 is the vibration strength of the shaker (apparently 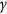 and *s* are constants). The rescaled data nearly collapse together along the dashed line, which indicates that rescaled fluxes are inversely proportional to the vibration frequency, that is, 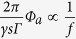. Apparently, the effective flux 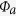 is proportional to 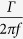 which is the peak velocity of the shaker, i.e., the effective flux is nearly independent of the frequency and vibration strength for a given value of the peak velocity. The conclusion is not surprising, provided that the peak velocity relates to the relative mean velocity of the granular pile to the sieve plate.

### Geometric effects of sieve plate on the flux

**Figure 1:**
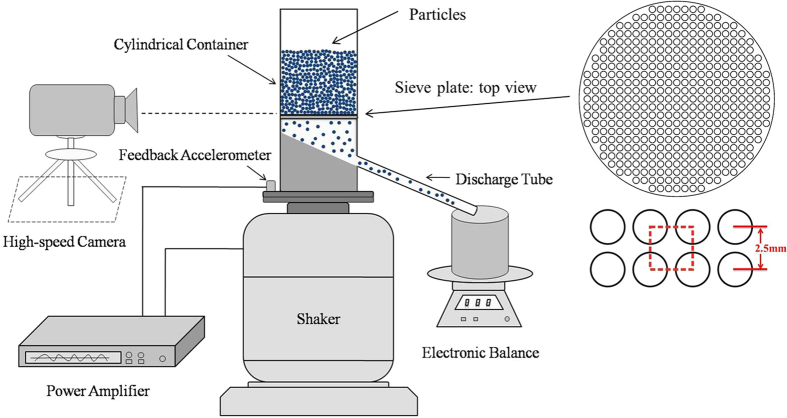
Schematic of the experimental establishment is shown. The enlarged portion exhibits a detailed geometry of the sieve plate.

**Figure 2:**
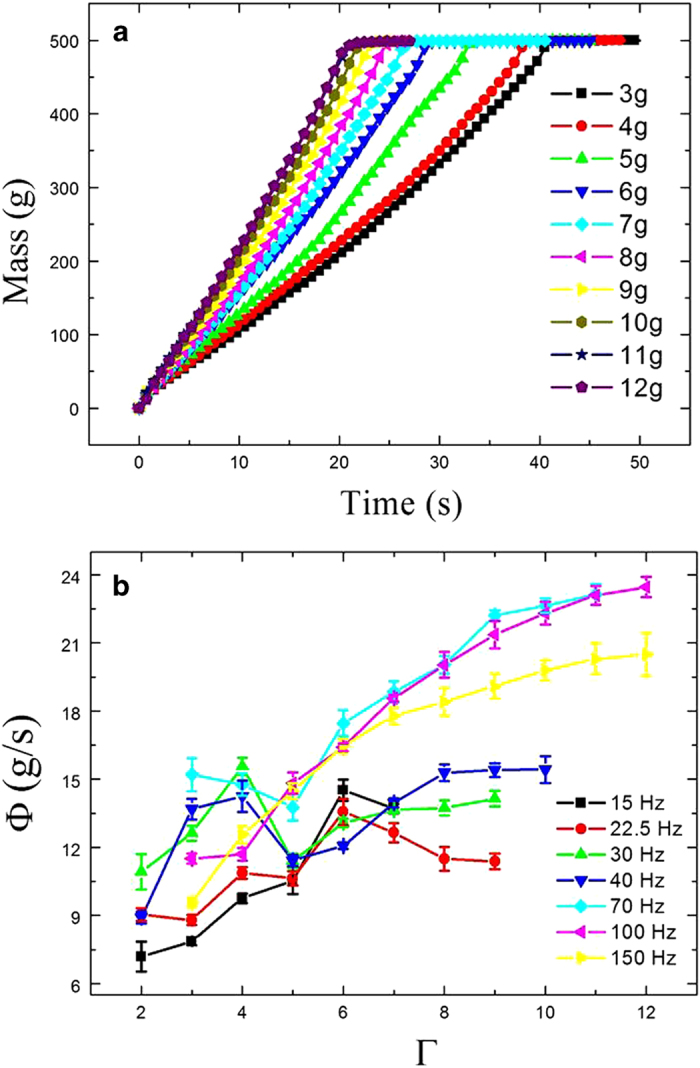
(**a**) Measured masses of the outflow as a function of time are shown at *f* = 100 Hz with different vibration strengths. These curves exhibit a nearly linear behavior except for the transient parts at the start and end of the flow. (**b**) Mean mass flux 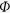 as a function of the vibration strength 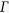 is shown. The relation between the flux and the vibration strength is rather complicated over the range of the vibration frequency.

**Figure 3:**
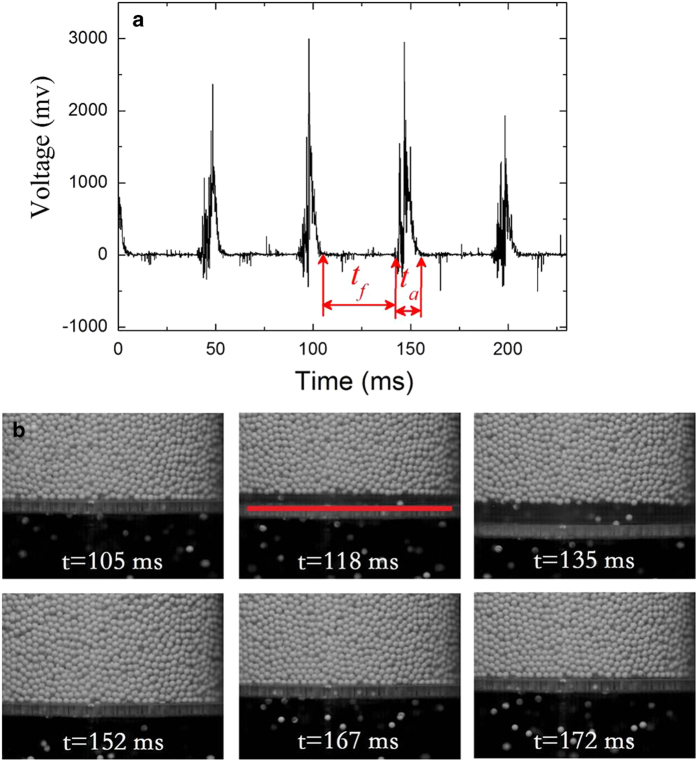
(**a**) Voltage signal measured by a pressure transducer is plotted as a function of time. 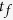 indicates the free flight time in which the pressure exerted by the granular pile vanishes. 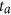 indicates the adhesion time when the granular pile contacts and moves together with the sieve plate. (**b**) A series of successive snapshots, photographed by a high-speed camcorder with the sample rate 600 frames per second, illustrate the motions of the particles bulk. *t* = 105 *ms*, the granular pile contacts with the sieve plate; *t* = 118 *ms*, the granular pile launches into air, detaching from the plate. No particles from the pile can pass through the upper surface of the sieve plate, which is indicated by a red, solid line; *t* = 135 *ms*; the granular pile rises highest in air; *t* = 152 *ms*, the granular pile falls down and just contacts the plate, the flow resumes; *t* = 167 *ms*, the granular pile stays on the plate, moving together with plate; *t* = 172 *ms*. The granular pile continues in contact, and the flow becomes more apparent. The vibration condition is *f* = 15 Hz, 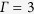.

**Figure 4:**
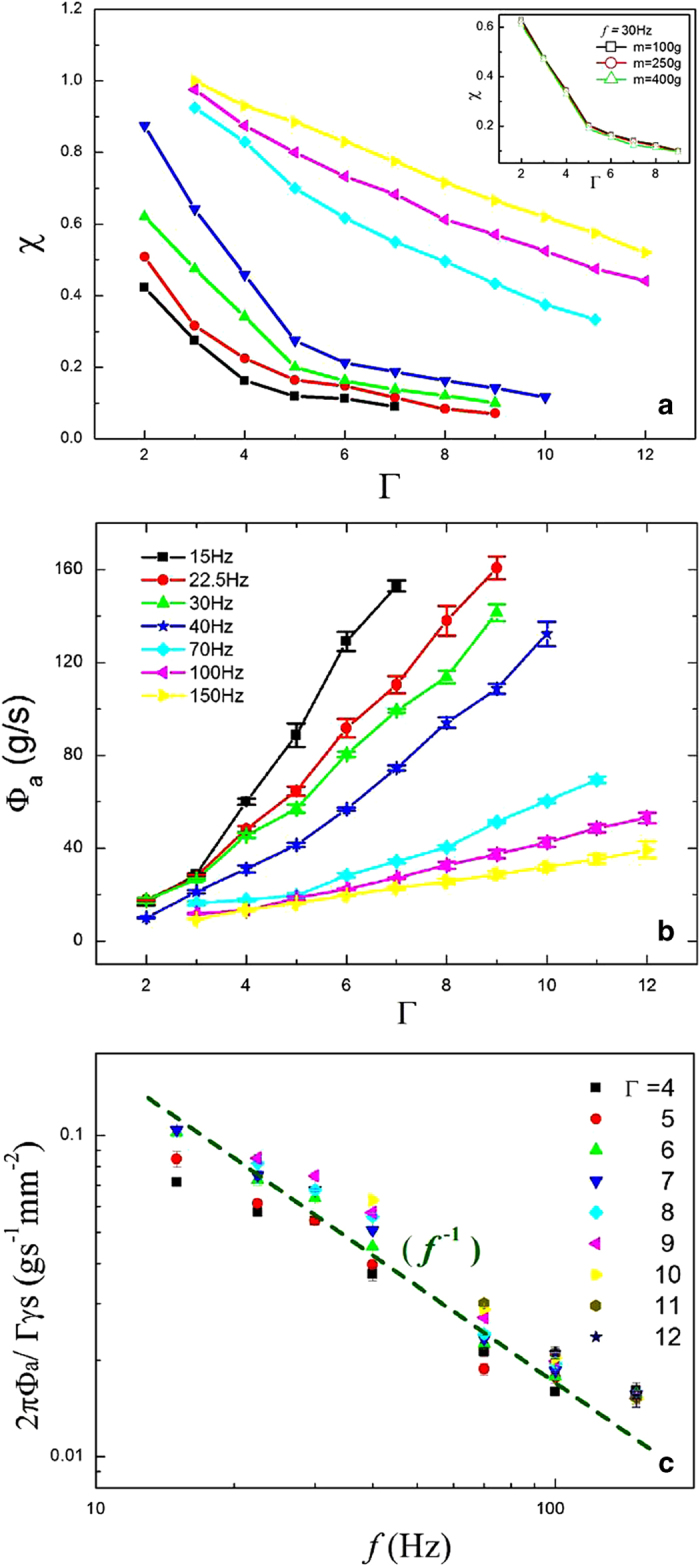
(**a**) Adhesion time fraction 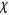 as a function of 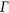 at a range of frequencies, (■) 15 Hz,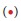 22.5 Hz,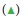 30 Hz,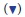 40 Hz, 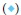 70 Hz,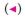 100 Hz,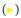 15s0 Hz, showing a monotonic decrease. Inset: the adhesion time fraction 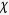 as a function of the vibration strength 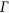 at *f* = 30 Hz as the discharging mass is 100 *g*, 250 *g* and 400 *g*, in order to examine the independence of the adhesion time fraction on the discharging mass. **(b**) Effective flux, 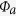, as a function of the vibration strength 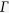 is shown over the range of the vibration frequency, showing a nearly linear fashion. (**c**) Rescaled flux is plotted as a function of the vibration frequency *f* for different vibration strengths 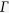 on a log-log scale. All rescaled curves approximately collapse together. The green dashed line 1/*f* is drawn to guide the eye.

**Figure 5:**
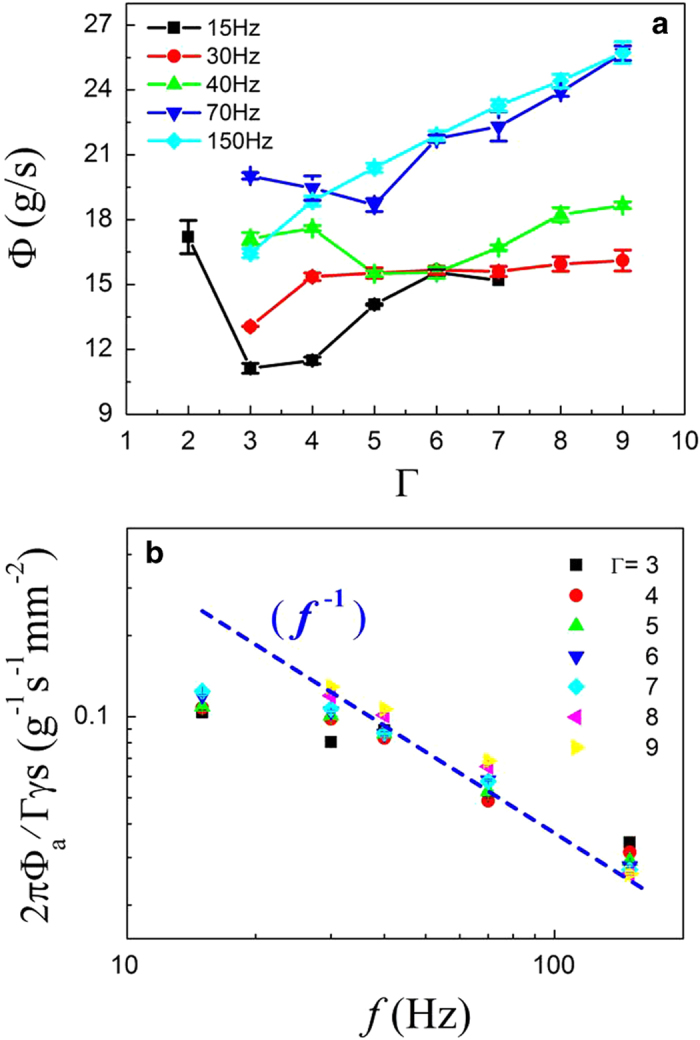
Granular particles flow through the plate on which sieve holes are arranged in the pattern of 2-D square grid with hole diameter 2.5 mm. (**a**) Mean mass flux 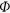 as a function of the vibration strength 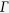 is shown. (**b**) Rescaled flux is plotted as a function of the vibration frequency *f* for different vibration strengths 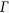 on a log-log scale. The blue dashed line 1/*f* is drawn to guide the eye.

**Figure 6:**
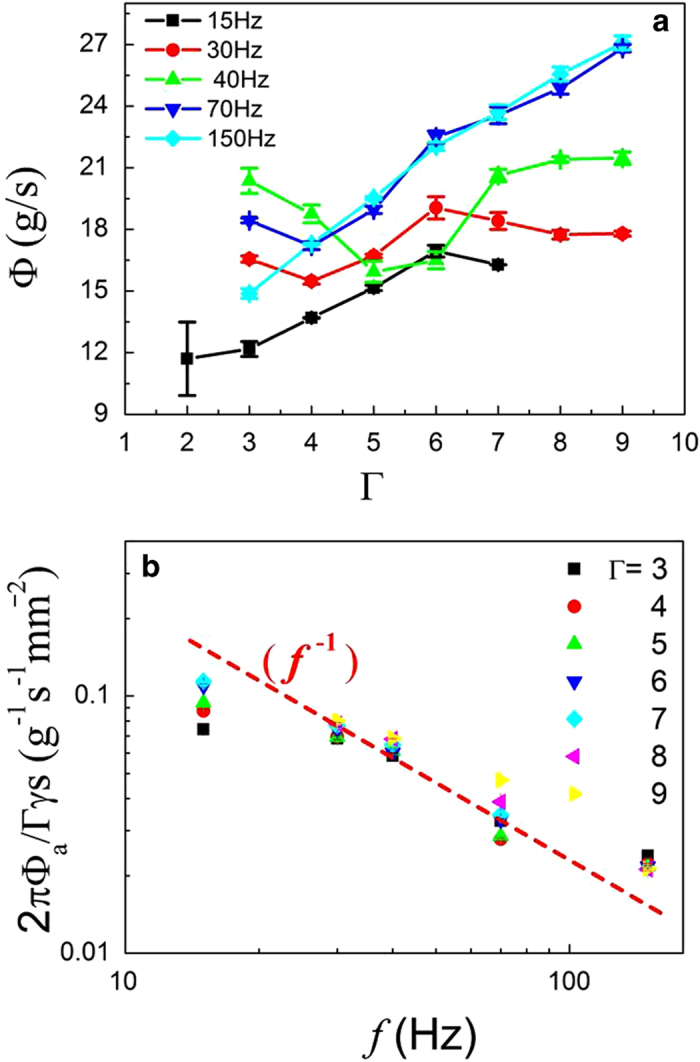
Granular particles flow through the plate on which sieve holes are arranged in the pattern of 2-D hexagonal grid with hole diameter 2.0 mm. (**a**) Mean mass flux 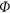 as a function of the vibration strength 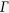 is shown. (**b**) Rescaled flux is plotted as a function of the vibration frequency *f* for different vibration strengths 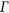 on a log-log scale. The red dashed line 1/*f* is drawn to guide the eye.

To consolidate the results obtained from the sieve plate in Fig. 1, we also examined two other sieves with different geometries. One arrangement pattern is also 2-D square grid, but with larger hole diameter 2.5 mm. The total number of the holes is 213, and the distance between the centers of two neighboring holes is 3.5 mm. Another arrangement pattern is a hexagonal grid (honeycomb grid) with the hole diameter 2.0 mm. The total number of the holes is 429 and the distance between the centers of two neighboring holes is 2.6 mm. We have examined the validity of the results with the two sieve geometries, and found that similar results persist (see Fig. 5 and Fig. 6). Itsuggests that the conclusions are robust and insentive to the geometry of the sieve plate.

Previous work mainly concentrated on the outflowing flux through a single, large hole, in which the granular flow stays constant even without the external vibration. If the hole can be reduced below the critical size, the flow will probably become intermittent, and namely in each vibration cycle one particle does not necessarily pass through the hole. In particular, both Chen *et al.* and Wassgren *et al.* studied the dependence of the discharging mean mass flux on the vibration strength and its frequency^24^,^25^. They found the outflowing flux decreases with increasing vibrating strength 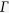, and proposed the outflowing flux under vibration can be described in the fashion of the empirical equation of Beverloo^25^. The experimental result is qualitatively different from ours. In our experiment, the relationship between the flux and the vibrating strength is considerably complicated, obviously showing a non-monotonic behavior for most situations. In addition, it looks impracticable to extrapolate the flux of such small holes from the Beverloo’s equation because it is only valid for the hole diameter larger than the critical size. Presumably, the disagreement results from the size of the holes and the flight time. We intend to explore these questions in future works by changing the size of holes, the geometric features of the sieve plate and vibrating conditions.

In summary, we first experimentally investigate the mean mass flux 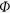 of the spherical particles through a sieve plate under vertical vibrations. This flux exhibits an intricate non-monotonic dependence on the vibration strength 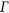. By using high-speed photography to reveal the motion of the particles near the sieve plate, it is found that the mass flow turns out to be an intermittent flow, and the whole flow process consists of the free flight and adhesion process. Since only the adhesion process contributes to the flux, we measure the adhesion time by two independent approaches, and define the adhesion flux 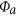 by introducing the adhesion time fraction 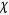. Then, we find a monotonic relation between the adhesion flux and the vibration strength. On the basis of these experimental results, we consider the vibration strength is supposed to influence the flux 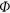 on both aspects: the enhancement of the fluidization of the granular pile increases the flux; the prolongation of the free flight time reduces the flux. The complexity of the mass flux 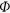 is due to the competition of the fluidization extent and the free flight duration of the granular pile. Finally, we discover that the adhesion flux is controlled by the vibration peak velocity of the shaker, and examine these conclusions with other sieves with different geometries.

## Methods

### Experimental set-up

A plexiglass cylindrical container is bolted on the platform of an electromagnetic shaker, which supplies vertical sinusoidal motion, with the ratio of the amplitude of the vertical vibration to that of the horizontal vibration less than 3%. An accelerometer attached to the shaker continuously monitors the vertical vibration. The motion of the shaker is conveniently characterized by the vibration frequency *f* and the dimensionless acceleration 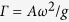, where *A* is the vibration amplitude, 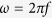 is the vibration angular frequency and *g* is the gravitational acceleration. In this experiment, we choose the frequency *f* from 15 Hz to 150 Hz and the acceleration 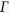 over the range of 2–12. The container of the diameter 60 *mm* (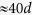, *d* is the diameter of a particle) is divided into two sections by a flat sieve plate. The upper section of the container is 150 *mm* high and is filled with 500 grams of alumina spheres, whose density and diameter are 2.7 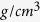 and 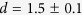 mm, respectively. The particle size is large enough so that the effects of interstitial air could be neglected. A base in the lower section slopes with an angle 34 ° to facilitate the collection of the particles passing through the sieve plate. A circular hole of diameter 30 *mm* is drilled on the container wall at the bottom of the base, and a discharging tube connected to the hole transports the outflowing particles to an electronic scale. The flat sieve plate (see the top view in Fig. 1) is 2 mm thick and 420 circular holes on the plate are arranged in the pattern of a 2-D square grid where the distance between the centers of two neighboring holes is 2.5 mm. The diameter of each hole is 2 *mm* (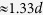), which is slightly larger than that of the alumina spheres, and thus more than one alumina sphere is not allowed to pass through a hole at the same time. The flow of the alumina particles through the sieve plate ceases when the container is at rest. Excited by an external vibration, the particles that fall through the holes flow into the discharging tube along the declining base, and then are weighed by an electronic scale with the precision 0.1 *g*, which is used to record the mass of the discharging alumina particles as a function of time, *m*(*t*). It is noteworthy that we have examined two durations for the whole flow: the duration I, the time from the start to the end of the particle flow via the sieve plate; and duration II, the time from the start to the end of the measurement of the electronic scale. The difference between the two durations is less than 1 s, which suggests that the flux measured by the electronic scale can characterize the mean flux through the sieve plate. Finally, to reveal the details of the flow process, we apply a high speed camcorder (Phantom V7.3) to observe the motion of the alumina particles near the sieve plate.

## Additional Information

**How to cite this article**: Wen, P. *et al.* Flux of granular particles through a shaken sieve plate. *Sci. Rep.* **5**, 9880; doi: 10.1038/srep09880 (2015).
